# Provenance of uranium particulate contained within Fukushima Daiichi Nuclear Power Plant Unit 1 ejecta material

**DOI:** 10.1038/s41467-019-10937-z

**Published:** 2019-06-26

**Authors:** Peter G. Martin, Marion Louvel, Silvia Cipiccia, Christopher P. Jones, Darren J. Batey, Keith R. Hallam, Ian A. X. Yang, Yukihiko Satou, Christoph Rau, J. Fred W. Mosselmans, David A. Richards, Thomas B. Scott

**Affiliations:** 1 Interface Analysis Centre, School of Physics, HH Wills Physics Laboratory, University of Bristol, Bristol, BS8 1TL UK; 2 Department of Earth Sciences, University of Cambridge, Bullard Laboratories, Madingley Road, Cambridge, CB3 0EZ UK; 3 Diamond Light Source, Harwell Science and Innovation Park, Didcot, OX OX11 0DE UK; 4 Collaborative Laboratories for Advanced Decommissioning Science, Japan Atomic Energy Agency, Tomioka-Machi, Futaba-gun, Fukushima, 979-1151 Japan; 5 School of Geographical Sciences, University of Bristol, Bristol, BS8 1SS UK

**Keywords:** Natural hazards, Nuclear waste, Characterization and analytical techniques, Characterization and analytical techniques

## Abstract

Here we report the results of multiple analytical techniques on sub-mm particulate material derived from Unit 1 of the Fukushima Daiichi Nuclear Power Plant to provide a better understanding of the events that occurred and the environmental legacy. Through combined x-ray fluorescence and absorption contrast micro-focused x-ray tomography, entrapped U particulate are observed to exist around the exterior circumference of the highly porous Si-based particle. Further synchrotron radiation analysis of a number of these entrapped particles shows them to exist as UO_2_—identical to reactor fuel, with confirmation of their nuclear origin shown via mass spectrometry analysis. While unlikely to represent an environmental or health hazard, such assertions would likely change should break-up of the Si-containing bulk particle occur. However, more important to the long-term decommissioning of the reactors at the FDNPP (and environmental clean-upon), is the knowledge that core integrity of reactor Unit 1 was compromised with nuclear material existing outside of the reactors primary containment.

## Abstract (web summary)

The larger particulates from reactor Unit 1 of the Fukushima Daiichi Nuclear Power Plant have received sparse attention compared to the Unit 2 particulate. Here the authors perform the higher-resolution and 3-dimentional analysis of several high-density micron-scale fragments, from within a larger Unit 1-derived representative ejecta particle.

## Introduction

Air-fall material derived from the Fukushima Daiichi Nuclear Power Plant (FDNPP) accident has formerly been isolated and analyzed from localities across Japan, extending hundreds of kilometers away from the facility^1–4^. In contrast to the smaller and highly spherical fallout particles observed at greater distances from the accident site, larger particulates extracted from bulk sediment material sourced from close to the facility with a complex exterior form^5,6^ here we reveal new insight into the accident conditions.

Following the March 2011 accident and radioactivity release, core inventory modeling using the ORIGEN source-code^7^ attributed specific radiocesium (Cs*) activity and atomic (isotope) ratios to each of the sites of three operational reactor Units (1, 2, and 3) and spent fuel storage ponds (1, 2, 3, and 4)^8^. These characteristic ratios arise due to the contrasting half-lives of the fission product isotopes—^137^Cs (*t*_1/2_ = 30.17 years) and ^134^Cs (*t*_1/2_ = 2.065 years)^9^, and also as a consequence of the differing levels of burn-up of the fuel at each of these localities. Nishihara et al.^8^ attributed a ^134^Cs/^137^Cs activity ratio >1.0 to material derived from reactor Units 2 and 3 (1.08 and 1.04, respectively) and values <1.0 (0.94) to that from reactor Unit 1. Resulting from the decay of the shorter-lived ^134^Cs, activity values from the spent fuel ponds, in contrast, were <1.0 (Unit 1: 0.54, Unit 2: 0.64, Unit 3: 0.74, and the most heavily loaded, Unit 4: 0.68).

Unlike the major north-west trending fallout plume (the source of the majority of the land-ward contamination) that has been shown to exhibit the characteristic Cs* signature of reactor Unit 2^10–12^, the Unit 1 release is of limited spatial extent. This contrasts with having not been released following the large-scale reactor building hydrogen explosions observed at Units 1 and 3^13^, but a believed breach in the structural integrity of the primary containment vessel (PCV) on the 15 March 2011 (following extensive, later inspections)^13–15^.

Alongside wide-area mapping and monitoring studies, single-particle analysis has confirmed that the individual fine-scale radioactive material sourced from the region to the north-west of the plant also exhibited a Cs* ratio attributable to the FNDPP Unit 2^16^. Owing to its widespread environmental dispersion, this particulate has been the subject of the majority of the Fukushima nuclear forensics studies undertaken thus far. Defined in the initial scanning electron microscopy (SEM) and energy-dispersive spectroscopy (EDS) particulate analysis of Adachi et al.^2^ as Cs-balls, these highly spherical and Si-based particles were collected by high-volume aerosol sampling in Tsukuba, 170 km SW of FDNPP. Kogure et al.^17^, conduce these Cs-balls to be formed by atmospheric condensation (around solid nucleation sites), however, attempts to reproduce such glassy particles has proved unsuccessful, which led the authors to suggest that the micro-particles were formed in highly specific conditions in the nuclear reactor environment. Subsequent synchrotron analysis of the same particulate by Abe et al.^1^, revealed the occurrence of U, in the center of a numbers of these particles—each of which averaged 2.1 μm in diameter. Further studies have extended the analysis of such micron-scale, reactor Unit 2-derived particulate through the application of destructive techniques, including transmission electron microscopy (TEM) following focused ion beam (FIB) sectioning to examine the interior structure and composition of the samples^3,4,17,18^.

Here we focus on the much larger diameter (>100 μm) and considerably more angular particulates collected much closer to the FDNPP^3,5,16^, which possess a lower ^134^Cs/^137^Cs activity ratio of <1.0 and are therefore ascribed the radioactive release from reactor Unit 1 (occurring earlier on the 12 March 2011, associated with the reactor-building hydrogen explosion^13–15^). The contamination (and particulate) was deposited within a plume to the north–north-west/north of the Fukushima site—trending towards the nearby city of Minamisoma—consistent with the prevailing wind conditions and directions at the time of the release^19,20^. In addition to the contrasting release mechanisms, the main 60 km long (Unit 2) contamination plume was the result of wet deposition caused by coincident rainfall events—whereas the plume from Unit 1 was deposited under dry conditions^21,22^. Corroboration of the spatially contrasting ^134^Cs/^137^Cs activity ratios around the FDNPP has been detailed in the work of Nishizawa et al.^11^.

The larger particulates have received sparse attention compared to the Unit 2 particulate, perhaps, because it has been found closer to the FDNPP site (<5 km), where people will not return to reside for a considerable period of time^23^. This Unit 1 material was first identified and isolated by Satou^16^ who identified a mean particle diameter >100 μm and a lower activity (Bq) per particle volume (μm^3^) (1.0 × 10^5^ × [volume]^0.39^) than Unit 2-derived material (2.0 × 10^16^ × [volume]^1.40^)^24^. Initial synchrotron characterization of this material, comparing its composition to the smaller Cs-balls was undertaken by Ono et al.^6^. Within this work, on a small 75 μm × 75 μm spherical portion of an elongate sub-mm particle, a highly heterogeneous (including Mo, Fe, Ni, Cd, Sn, and Cr) distribution of elemental constituents was identified alongside the existence of U in addition to other elements concentrated around its surface. Additional analysis of Unit 1 particulate suggests that the bulk of the sub-mm particle results from the melting and amalgamation of fibrous (Si-based) Rockwool™ thermal insulation material under the intense heat following the loss of coolant incident (LOCI)^24^.

This work seeks to determine if these inclusions (that have same length-scale as the U particulate contained within the highly spherical Unit 2 particulate^1^), are of actinide composition and if so, whether they can be unequivocally sourced to Unit 1 at the FDNPP. The means of attributing the U to specific FDNPP reactors relies on contrasting ^235^U-enrichment levels contained in the different reactors fuel assemblies. While reactor Units 2 and 3 contained fuel with 3.8 wt% ^235^U (greater than the 0.7 wt% ^235^U of natural U), the fuel of Unit 1 was constituted by ^235^U concentrations of between 3.4 and 3.6 wt%^25^.

## Results

### Fluorescence tomography

**Fig. 1:**
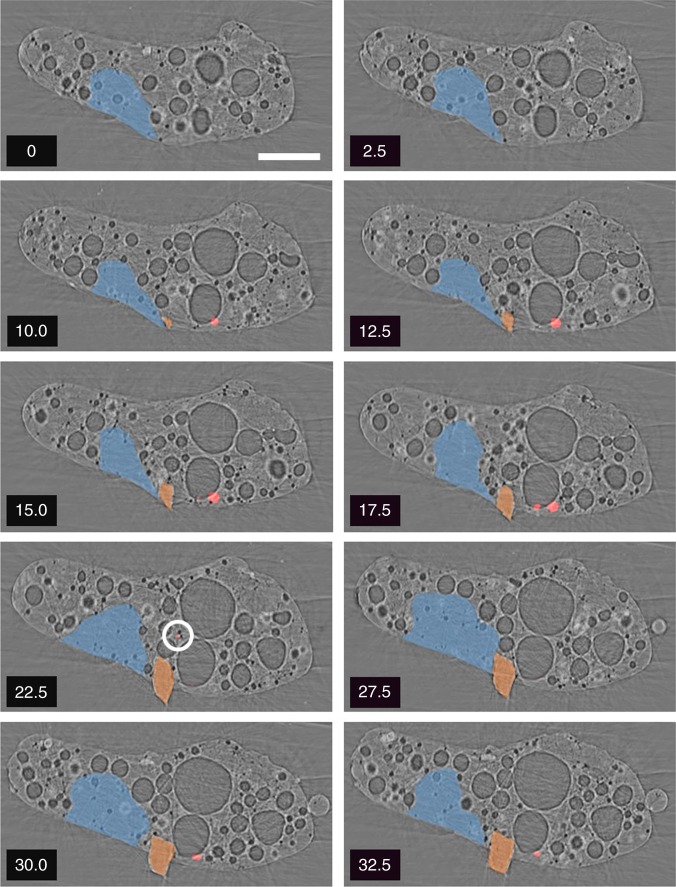
Combined X-ray tomography and fluorescence cross-sections: sequential longitudinal slices (upwards from the horizontal mid-plane) through the SR-μ-XRT reconstruction, overlain with U composition data (shown in red, and additionally circled in 22.5 µm section) as determined via SR-μ-XRF mapping. The location of Fe (orange) and cement (blue) composition regions are highlighted. The values shown represent the thickness of the tomograph

Rendering of the U signal derived from synchrotron radiation micro-x-ray fluorescence (SR-μ-XRF) applied to a series of 2.5 μm thickness slices obtained following the synchrotron radiation micro-x-ray tomography (SR-μ-XRT) analysis are shown in Fig. 1. As a result of the earlier compositional analysis performed on this material, the locations of Fe-rich and cement fragments contained within this material are also shown. From these two-dimensional reconstructions, the U particulate is observed to be near-exclusively associated with the exterior circumference of the particle—occurring at depths averaging 10 μm into the highly porous Si-based matrix. While the U particulate appears to possess a highly rounded form (owing to the 5.0 μm diameter round beam profile and resulting 2.5 μm step size of the XRF measurements) the size and spherical shape is likely to represent an exaggeration of its true size and a greater degree of rounding than its true form.

We note that one U particle is found not on the particulates exterior, but rather within the sub-mm CF-01 sample. This particle (highlighted in Fig. 1g—22.5 μm thickness section) is conversely enclosed by several spherical voids (that together constitute 24% of the particle’s total internal pore volume), in addition to being closely associated with the highlighted Fe and cement composition fragments. Following the earlier examination of this ejecta material, such a high-energy release scenario is believed to have embedded these reactor-sourced structural fragments (such as steel and cement) into the surface of this Si-based insulation material. The incorporation of these fragments into this softened material resulted in the U particulate, formerly located around its surface, becoming pushed deeper into the particle towards its center. The existence of the larger number of more peripheral U particles (located only several microns under the bulk particles surface) can be attributed to the softened state of the Si-based matrix combined with the considerable gas (volatile) over-pressure that existed around the reactor’s PCV environment. These conditions served to force the actinide composition material into the particle, having been generated by the earlier U volatilization/particle formation that followed the integrity compromise and extensive melting of the Unit 1 reactor core^13,14^.

### X-ray absorption near edge structure

**Fig. 2:**
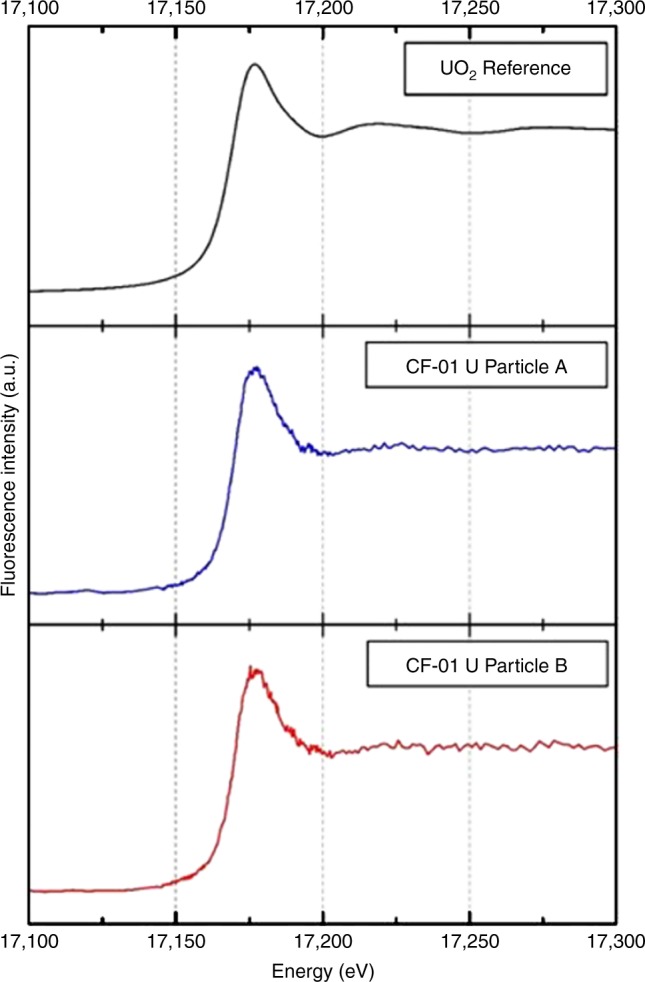
X-ray absorption edge profiles: SR-μ-XANES fluorescence intensity plots derived from two of the U composition particles contained within the sub-mm Si-based particle, alongside that of a comparison reference UO_2_ spectra, from^44^

The results of the analysis performed on two of these inclusions are presented in Fig. 2. The x-ray absorption near-edge structure (XANES) spectra of the particles are characterized by a broad white line that peaks at 17,176–17,177 eV, before smoothly decreasing to a minimum at 17,200 eV. This shape has been previously attributed to the U(IV) oxidation state in uranium oxides and glasses, whereas more oxidized forms, such as U(V) and U(VI), are characterized by an increasingly asymmetric shape, with an additional shoulder growing around 17,185–17,195 eV^26–28^.

Beam damage, characterized by the reduction of the white line intensity within tens of seconds, has previously been reported for U-containing glasses by Halse^29^. However, these modifications are not accompanied by further changes of the XANES shape relating to a change in the oxidation state upon exposure to the incident x-ray beam. Here, spectra remain unchanged after 15 min of continued beam exposure and data collection—therefore discounting any beam-induced oxidation of the particles. While it is possible to obtain XANES data from the U-containing particles, local structure information derived from the succeeding EXAFS region of the spectra is not amenable in this instance, this likely the result of the large size of the incident x-ray beam (2 μm × 2 μm) in comparison to that of the enclosed U particulate, the subject of analysis.

These results confirm the identical structure of the particulate contained within the CF-01 sample to that of standard UO_2_ nuclear fuel. While highly suggestive of the high melting-point fuel material used extensively in nuclear reactors around the world, U in the (IV) state is also found in numerous naturally occurring primary and secondary uranium ore minerals^30^—with one of the most commonly encountered being that of uraninite (UO_2_). With the Si-based particle’s precursor Rockwool™ insulation material (derived from a basaltic precursor material) typified by a low U content of 0.2 ppm^31^, and owing also to the spatially heterogeneous (circumferential) distribution of the U particulate, an anthropogenic provenance is most likely. It is therefore also through the application of SIMS to derive true isotopic ratios that a natural occurrence can be entirely excluded.

### Secondary ion mass spectrometry

**Fig. 3:**
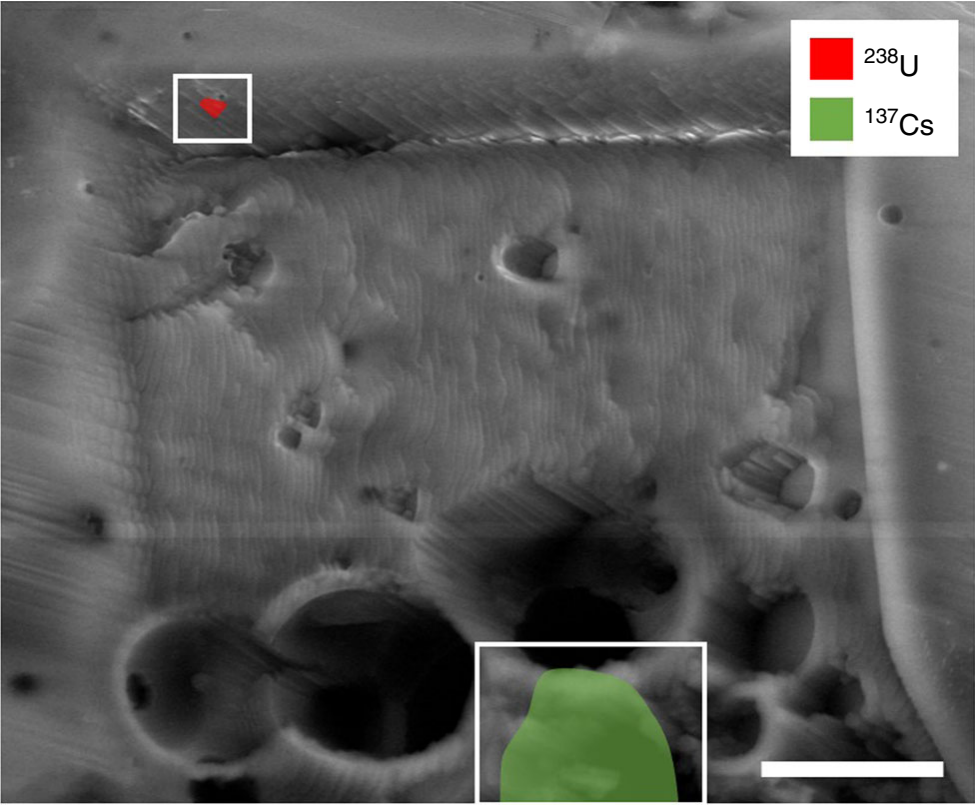
SIMS depth-profile compositional mapping: compositional mapping results (^238^U and ^137^Cs) overlain onto the trench produced following SIMS depth-profiling into FIB-cut face. Scale bar = 10 μm

With SR-μ-XANES analysis showing the U to exist in the U(IV) oxidation state (as UO_2_) and, therefore, the composition of either nuclear fuel or naturally occurring mineral material, the isotopic results provided by SIMS analysis serves as the critical indicator in ascribing it definitively to a reactor source—and to Unit 1 at the FDNPP. The result of SIMS compositional mapping over the vertical cut face produced by ion beam depth profiling is shown in Fig. 3. From this image, a micron-scale particle at 238 amu (marked in red) is observed on the vertical face of the milled region, as expected following earlier XRF elemental mapping and ion beam sample preparation. Also apparent is a discrete region of ca. 10 μm diameter at the base of the trench, with a mass of 137 amu—attributed to the fission product ^137^Cs.

**Fig. 4:**
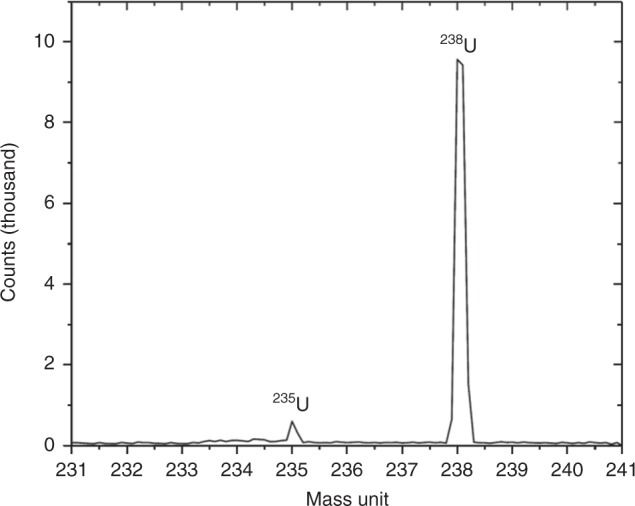
Uranium mass spectra: mass spectra between 231 and 241 amu obtained from the U particle contained within the CF-01 bulk particle (as identified in Fig. 3)

The mass spectra (between 231 and 241 amu) of this U particle is shown in Fig. 4. From this, the discernible peaks are at 238 and 235 amu. The ratio of integral peaks gives an atomic ratio (^235^U/^238^U) of 0.0354 ± 0.0015 (3.54 ± 0.15 wt% ^235^U). This elevated ^235^U concentration above the globally averaged natural abundance of 0.72%^32^ clearly identifies this material as being anthropogenic. The absence of other masses (e.g. 234, 236, 239, and 240 amu) within the spectra (Fig. 4) could be attributed to the lack of fuel burn-up of this component of fuel material, and therefore transmutation of the parent isotopes that would result in the ingrowth of these additional mass species (N.B. the burn-up of the fuel in reactor Unit 1 averaged a considerable 39.5 to 45 GWD/t). The poor mass-sensitivity of SIMS at these higher mass-units could equally be invoked to represent the detection of only these two masses (^235^U and ^238^U), and not the lower concentrations of the other actinide species that may exist. With higher-sensitivity instrumentation, mass peaks at both 234 and 236 amu would be expected—lending additional support to the materials reactor provenance.

While the Cs (137 amu) region is characteristically rounded in its form, the U fragment is significantly more angular. This angularity supports the theory that a loss of structural integrity occurred in the fuel assemblies following their extensive melting in the LOCI and an ensuing fragmentation/particle generation occurred during the subsequent reactor building hydrogen explosion^13–15^.

In contrast, the more spatially diffuse distribution exhibited by the Cs (Fig. 4) invokes a differing provenance to that of U. This can be ascribed to the known difference in the melting point/volatilization temperature of the two elements^33^. At the time of the accident, the highly volatile fission product (Cs), as well as other similarly volatile elements, would have existed in a gaseous state at a considerable over-pressure within the reactor Unit 1 pressure vessel. This gas was resultantly incorporated into the partially molten Si-based material in the diffuse manner observed.

**Fig. 5:**
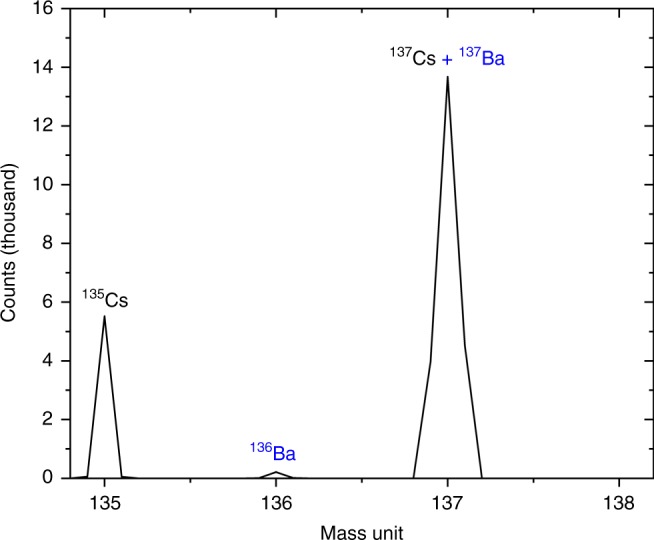
Cs and Ba mass spectra: SIMS mass spectra (positive bias) between 135 and 138 amu, derived from the Cs-rich region evidenced to exist within the CF-01 bulk particle (identified in Fig. 3)

As the two primary radioisotopes of cesium (^134^Cs and ^137^Cs) decay to stable isotopes of barium (^134^Ba and ^137^Ba, respectively), an inventory of Ba would exist associated with this Cs-rich region as a result of radiogenic ingrowth. The secondary ion mass spectra (using a positive voltage bias) over the 135–138 amu mass window is shown in Fig. 5. This spectra comprises two mass peaks; 135 and 137 amu. The mass peak at 135 amu is likely to represent the sole contribution from ^135^Cs, a long-lived fission product (*t*_1/2_ = 2.3 × 10^6^ years). In contrast, the mass peak at 137 amu is a combination of ^137^Cs, alongside a minor contribution from radiogenic Ba. As well as this ingrown Ba, a further source of the element is that which arises from the naturally occurring Ba. However, such a contribution from pre-existing (natural) Ba in this instance is precluded because of the absence of a mass peak at 138 amu—the primary mass of Ba, therefore suggesting that this Ba results entirely from radiogenic ingrowth. The small contribution at mass 136 amu is the likely result of the decay of the short-lived ^136^Cs (*t*_1/2_ = 13.16 days) into the stable ^136^Ba.

### Source attribution

**Fig. 6:**
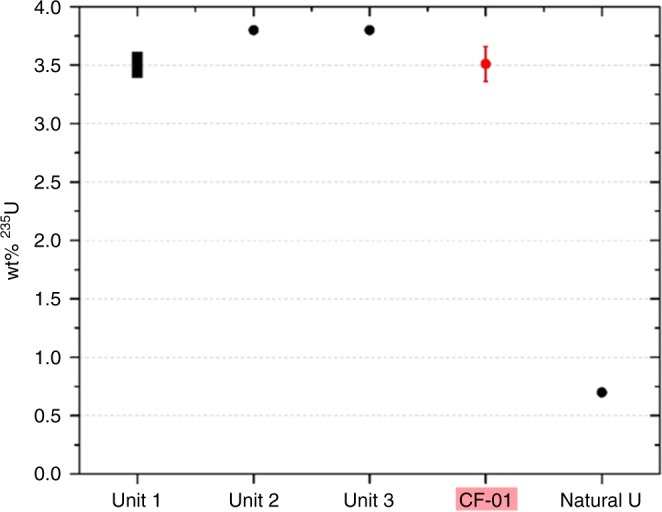
Core inventory comparison: comparison of the ^235^U wt% (±2*σ*) measured in this work via SIMS (CF-01) with operational wt% ^235^U values published by the sites operator, TEPCO^25^

With the likely anthropogenic provenance of the U particulate inclusions shown through combined SR-μ-XANES and SIMS analysis, a comparison of the ^235^U content of this U particle (CF-01) is shown alongside the published values for reactor Units 1, 2 and 3 (alongside that of natural U) in Fig. 6. Having been attributed in earlier works to reactor Unit 1 through its ^134^Cs/^137^Cs activity ratio^16^, the atomic ratio ^235^U/^238^U content in this single U particle further supports this provenancing. In contrast to reactor Units 2 and 3 which, as shown in Fig. 6, were operating with higher UO_2_ fuel enrichments of 3.8 wt% ^235^U, Unit 1 was operating with enrichments between 3.4 and 3.6 wt% ^235^U. The 3.54 ± 0.15 wt% ^235^U (0.0354 ± 0.0015 ^235^U/^238^U atomic ratio) determined for the (CF-01) particle reported here is, therefore, consistent with the published core loading values for reactor Unit 1.

## Discussion

We detail the first multi-technique evaluation on U-containing material sourced from the reactor building hydrogen explosion that occurred in Unit 1 of the FDNPP. The combined use of advanced synchrotron-based x-ray analysis techniques alongside the isotopic analysis afforded by SIMS, shows that the U inclusions were isotopically enriched in ^235^U. Combining this result with the particles radiocesium ratio, we definitively attribute the sub-mm ejecta particle as having originated from reactor Unit 1. Any suggested natural provenance of this U (arising from species initially contained within the source Rockwool™ insulation material) can, therefore, be disregarded. Embedded within the glassy Si-based bulk particle (akin to vitrified waste^34^) and considering the insoluble nature of the U(IV) oxide material, a long-term environmental stability is implied—with a very-limited risk to health unless the bulk material were to fragment (e.g. through mechanical attrition). Despite any potential inherent toxicity of U being suppressed with the material in its current form^35,36^, considerations must be made to account for the likely existence of other (equally, if not more, toxic) actinide species (for example Pu), that may also exist within such Unit 1 ejecta material^37^. While the materials toxicity may not currently be of significance, this work has highlighted the extent of reactor core and containment failure at Unit 1 and the resulting implications for the long-term decommissioning strategy of the reactor core that is set to commence shortly. Although an inventory of other (longer-lived) fission product and activation species were released and could be identified utilizing the methods described in this work, this study has chosen to focus solely on source attribution using combined U and Cs analysis. Future work will utilize other elements and isotopic ratios to further constrain the materials provenance and conditions/environment of formation.

## Methods

### Sample collection and preparation

Bulk (~100 g) exposed samples, identified to exhibit elevated levels of radioactivity when scanned using a hand-held Geiger–Müller instrument, were collected in October 2014 within the highly contaminated Restricted Zone and a site formerly attributed to have been contaminated by reactor Unit 1^11,12^. This material was obtained from the ground (as dust samples) at 37.4379°N, 141.0222°E, 2 km NNW from the center of FDNPP. To extract the particles from the surrounding organic sediment, a multi-stage autoradiography, division, and extraction process was employed^38^. After removal, each of the sub-mm particles was placed onto a piece of adhesive Kapton™ film. An initial quantification of each particle was then performed using SEM with associated energy dispersive spectroscopy (EDS) and gamma-ray spectrometry using a high-volume detector, the results of which are discussed elsewhere^24^. Greater than 20 similar sub-mm particles were extracted from the sediment and found upon here representative of the average size/volume, with typical surface composition and internal structure^5^.

To enable SR analysis of this representative particle, it was further enclosed within additional layers of x-ray transparent Kapton™ film for the multiple synchrotron-based techniques. A corner of the Kapton™ envelope was glued onto a 1 cm aluminum support pin, which was then attached to a magnetic base for its installation and movement on the different synchrotron beamline stages. During this work, a representative CF-01 particle was used—with dimensions; 450 × 280 × 250 μm.

### Synchrotron radiation analysis

SR analysis of the particle was performed on the I13-1 (coherence imaging) and the I18 (micro-focus spectroscopy) beamlines at the Diamond Light Source (DLS; Harwell, UK)^39^. Both three-dimensional SR-μ-XRF and SR-μ-XRT of the particle were first performed on the I13-1 beamline, prior to SR-μ-XAS on the I18 beamline. With a distance of 250 m between the insertion device (canted undulator) and the sample, the I13-1 beamline utilizes the highly coherent x-rays produced by the experimental optics, with an energy range of 4–23 keV (a maximum of 19 keV was used in this work) and typical flux of 10^9^ photons/s. Despite being located closer to the insertion device, the I18 beamline uses a similar optical setup to the longer I13-1 beamline—with a comparable energy range of 3–22 keV, tunable in 0.5 eV increments using a cryogenically cooled Si-111 monochromator.

Using the I13-1 beamline at the Diamond Light Source^40^, the particle was studied using both μ-XRT, and 3D μ-XRF. For the XRT acquisition, the sample was illuminated with a 19 keV collimated X-ray beam. The particle was rotated over a range of 180° in steps of 0.1°. The projections were acquired with an optical microscope coupled to a 26 μm-thick GGG:Eu scintillator. The total optical magnification of ×20 provided a pixel size of 0.45 μm. For the 3D XRF analysis, the X-ray beam was formed through a 5 μm pinhole positioned upstream of the sample. At each angle of the 3D XRF, the sample was scanned with respect to the beam in a raster grid of 40 × 20 steps with a step size of 2.5 μm, giving a field of view of 100 μm × 50 μm. At each scan position, the X-ray fluorescence spectrum was acquired using a single channel silicon drift Vortex^®^ X-ray detector placed in the plane of the sample, normal to the direction of propagation of the beam. The sample was rotated over a range of 180° in steps of 4.5°.

From the series of fluorescence projections, the three-dimensional volume for each element was reconstructed using the ordered-subset penalized maximum-likelihood algorithm, with weighted linear and quadratic penalty algorithms in the TomoPy framework^41^. An iterative algorithm to correct for the degree of sample self-absorption that occurred was also employed for the tomography results. The reconstructed overlay images were produced using ImageJ and Python software platforms.

For the I18 SR-μ-XANES analysis, an identical particle setup was used. Prior to the analysis, an initial two-dimensional SR-μ-XRF scan was made of the particle at 21 keV (full beam size flux at 10 keV = 10^12^ photons/s), with the sample similarly rastered through the incident X-ray beam in 2 μm steps (resulting in a 2 μm spatial (pixel) resolution) to re-establish the positions of the high-density (U-containing) particles. Once these positions were located, SR-μ-XANES was performed. For this analysis, the stage position was maintained whilst the incident beam energy was varied—tuned by the monochromator over the U-L_III_ edge (17,166 eV), at various energy steps (5 eV from 17,025 to 17,140 eV, 0.5 eV from 17,140 to 17,205 eV, 1 eV from 17,205 to 17,275 eV, 2 eV from 17,275 to 17,400 eV and finally in 2.5 eV steps from 17,400 to 17,510 eV). Three repetitions of each scan were made in each instance to improve the resulting signal-to-noise ratio in the data. The SR-μ-XANES data was analyzed using the Demeter (ATHENA/ARTEMIS) suite of software^42^—based on the open-source IFEFFIT code^43^. Calibration of the I18 monochromator (with an inherent stability of ±0.05 eV per day) before and after the analysis was undertaken using a series of material standards with characteristic emission energies. The reference material standard for the U-L_III_ edge comparison was sourced from the International X-ray Absorption Society Database (Reference: atm-1c-glass reference l3 edge)^44^.

### Secondary ion mass spectrometry

Having determined the locations at which U composition particulate was located inside the parent particle, isotopic analysis was performed using magnetic sector (MS) SIMS. The MS-SIMS system used in this study was a custom-built instrument utilizing a primary Ga^+^ ion beam to sputter the samples surface before filtering and measuring the resulting secondary ion species across the 0–300 amu mass range. However, as the U-containing particulate was observed to exist several microns below the surface of the sample, FIB milling (using an FEI Helios NanoLab F600i dual FIB-SEM system) was conducted to expose the U particle and produce a cut face ~80 × 80 μm. Prior to this preparation (and subsequent SIMS analysis), the particle was carefully removed from its Kapton™ film envelope and placed onto a low elemental background electrically conductive carbon Spectro Tab (TED PELLA Inc.) before being sputter-coated (Edwards Vacuum Scancoat Six) with a 2 nm film of conductive Au. Progressively decreasing Ga^+^ ion beam cutting energies (reduced from 16 nA to 90 pA) were used to obtain a surface free from the artefacts of the milling process.

With a flat surface produced on the highly textured CF-01 particle to a depth just above the location of the U composition particle, depth profiling was first used to locate the edge of the actinide fragment. A ×5000 magnification was used—producing a 40 × 40 μm trench (alongside a dwell-time of 2500 ms for each of the six mass units measured) to mill to a depth of 10 μm. A rapid-scan ion map (two raster frames) was then performed on the cut vertical surface, where the U particle was observed to exist (using the same parameters as those employed for depth profiling) to exactly locate the contained particle. Finally, a spectrum over the U mass window (231–241 amu, with 0.05 amu steps and 5000 ms dwell-time) was performed to evaluate the specific isotopic composition of the U inclusion. This mass range was swept three times to enhance the signal quality and reduce spurious noise within the data.

## Data Availability

The raw (unprocessed) data that supports the findings of this study are available from Mendeley Data, with the 10.17632/46db2h9kwr.1
